# Methodologies and key considerations for implementing the International Classification of Diseases-11th revision morbidity coding: insights from a national pilot study in China

**DOI:** 10.1093/jamia/ocae031

**Published:** 2024-03-01

**Authors:** Meng Zhang, Yipeng Wang, Robert Jakob, Shanna Su, Xue Bai, Xiaotong Jing, Xin Xue, Aimin Liao, Naishi Li, Yi Wang

**Affiliations:** Department of Medical Records, Peking Union Medical College Hospital, Chinese Academy of Medical Sciences & Peking Union Medical College, Beijing 100730, China; Collaborating Center for the WHO Family of International Classifications in China, Beijing 100730, China; National Center for Quality Control of Medical Records, Beijing 100730, China; Collaborating Center for the WHO Family of International Classifications in China, Beijing 100730, China; Department of Surgery, Peking Union Medical College Hospital, Chinese Academy of Medical Sciences & Peking Union Medical College, Beijing 100730, China; World Health Organization, Geneva 1211, Switzerland; Department of Medical Records, Peking Union Medical College Hospital, Chinese Academy of Medical Sciences & Peking Union Medical College, Beijing 100730, China; Collaborating Center for the WHO Family of International Classifications in China, Beijing 100730, China; National Center for Quality Control of Medical Records, Beijing 100730, China; Department of Medical Records, Peking Union Medical College Hospital, Chinese Academy of Medical Sciences & Peking Union Medical College, Beijing 100730, China; Collaborating Center for the WHO Family of International Classifications in China, Beijing 100730, China; National Center for Quality Control of Medical Records, Beijing 100730, China; Department of Medical Records, Peking Union Medical College Hospital, Chinese Academy of Medical Sciences & Peking Union Medical College, Beijing 100730, China; Collaborating Center for the WHO Family of International Classifications in China, Beijing 100730, China; National Center for Quality Control of Medical Records, Beijing 100730, China; Department of Medical Records, Peking Union Medical College Hospital, Chinese Academy of Medical Sciences & Peking Union Medical College, Beijing 100730, China; Collaborating Center for the WHO Family of International Classifications in China, Beijing 100730, China; National Center for Quality Control of Medical Records, Beijing 100730, China; Department of Medical Records, Peking Union Medical College Hospital, Chinese Academy of Medical Sciences & Peking Union Medical College, Beijing 100730, China; Collaborating Center for the WHO Family of International Classifications in China, Beijing 100730, China; National Center for Quality Control of Medical Records, Beijing 100730, China; Department of Medical Records, Peking Union Medical College Hospital, Chinese Academy of Medical Sciences & Peking Union Medical College, Beijing 100730, China; Collaborating Center for the WHO Family of International Classifications in China, Beijing 100730, China; Department of Endocrinology, Key Laboratory of Endocrinology of National Health Commission, Peking Union Medical College Hospital, Chinese Academy of Medical Sciences & Peking Union Medical College, Beijing 100730, China; Department of Medical Records, Peking Union Medical College Hospital, Chinese Academy of Medical Sciences & Peking Union Medical College, Beijing 100730, China; Collaborating Center for the WHO Family of International Classifications in China, Beijing 100730, China; National Center for Quality Control of Medical Records, Beijing 100730, China

**Keywords:** ICD-11, morbidity coding, pilot study, coding accuracy, intercoder reliability, implementation

## Abstract

**Objective:**

The aim of this study was to disseminate insights from a nationwide pilot of the International Classification of Diseases-11th revision (ICD-11).

**Materials and methods:**

The strategies and methodologies employed to implement the ICD-11 morbidity coding in 59 hospitals in China are described. The key considerations for the ICD-11 implementation were summarized based on feedback obtained from the pilot hospitals. Coding accuracy and Krippendorff’s alpha reliability were computed based on the coding results in the ICD-11 exam.

**Results:**

Among the 59 pilot hospitals, 58 integrated ICD-11 Coding Software into their health information management systems and 56 implemented the ICD-11 in morbidity coding, resulting in 3 723 959 diagnoses for 873 425 patients being coded over a 2-month pilot coding phase. The key considerations in the transition to the ICD-11 in morbidity coding encompassed the enrichment of ICD-11 content, refinement of tools, provision of systematic and tailored training, improvement of clinical documentation, promotion of downstream data utilization, and the establishment of a national process and mechanism for implementation. The overall coding accuracy was 82.9% when considering the entire coding field (including postcoordination) and 92.2% when only one stem code was considered. Krippendorff’s alpha was 0.792 (95% CI, 0.788-0.796) and 0.799 (95% CI, 0.795-0.803) with and without consideration of the code sequence, respectively.

**Conclusion:**

This nationwide pilot study has enhanced national technical readiness for the ICD-11 implementation in morbidity, elucidating key factors warranting careful consideration in future endeavors. The good accuracy and intercoder reliability of the ICD-11 coding achieved following a brief training program underscore the potential for the ICD-11 to reduce training costs and provide high-quality health data. Experiences and lessons learned from this study have contributed to WHO’s work on the ICD-11 and can inform other countries when formulating their transition plan.

## Background

The International Classification of Diseases (ICD)-coded data have been widely used in health statistics, public health surveillance, health care performance evaluations, and reimbursement.^1^^,^^2^ The 11th revision of the ICD (ICD-11) is the latest international standard for health information; it was adopted at the 72nd World Health Assembly in 2019 and officially took effect on January 1, 2022.^3^ The ICD-11 is a modern, integrated classification and terminology system, rooted in current medical science and ontology-based content model.^4^ It enables the retention of fine-grained clinical diagnosis information in clinical records utilizing uniform resource identifiers (URIs) and provides additional details with postcoordination. With the advancement of informatics technology and increasing availability of informatics infrastructures, the ICD-11 was designed as an entirely digital product.^2^^,^^5^^,^^6^ Implementation of the ICD-11 offers new opportunities (and new challenges) in the collection, exchange, and analysis of health information, and aims to provide high-quality multidimensional data to improve health care and make the best use of health resources.

Since the initial release of the ICD-11 for Mortality and Morbidity Statistics (ICD-11 MMS) in June 2018, World Health Organization (WHO) member states and researchers have undertaken evaluations of its practical use.^6–9^ Following the completion of the Chinese translation of the ICD-11 in 2018, the National Health Commission of the People’s Republic of China endorsed the transition to the ICD-11.^10^ Subsequently, the Collaborating Center for the WHO Family of International Classifications in China developed an ICD-11 coding software, mapped the ICD-10 Chinese Modification to the ICD-11, and conducted a single-center pilot study of the ICD-11 morbidity coding at the Peking Union Medical College Hospital (PUMCH). Led by the research group of the present study, the single-center pilot study established a workflow for implementing the ICD-11 in morbidity coding, trained a group of trainers, refined the ICD-11 coding software, and developed educational materials.

While a large-scale pilot study is integral to the transition to the ICD-11, no study to date has reported on the ICD-11 morbidity coding at a regional or national level. In this article, we aimed to describe the strategies and methodologies used to implement a nationwide pilot study of the ICD-11 in morbidity coding, report the coding accuracy and intercoder reliability of clinical coders from pilot hospitals after participating in an ICD-11 training program, share the experiences and lessons learned, and discuss the key considerations in the transition to the ICD-11.

## Methods

### National pilot study

The national program piloting the use of the ICD-11 in morbidity coding was initiated by the Department of Medical Administration, National Health Commission of the People’s Republic of China in December 2021. A total of 59 tertiary public hospitals, including 35 general hospitals and 24 specialty hospitals from 31 provincial-level administrative regions (provinces, municipalities, and autonomous regions) and Xinjiang Production and Construction Corps in China, were nominated by the provincial health commissions based on their status as leading hospitals in their respective regions with robust IT infrastructure and supporting resources. A focal point responsible for coordinating the pilot study was designated by each pilot hospital. PUMCH was one of the pilot hospitals. The WHO-FIC Collaborating Center, which is affiliated with PUMCH, was entrusted to lead the program. This study excluded Traditional Medicine hospitals due to a concurrent pilot program. This separate program, led by the Center for International Classification Research on Traditional Medicine Clinical Conditions and Service Evaluation in China, focuses on an expanded version of the ICD-11 Traditional Medicine Conditions Module I.

All pilot hospitals were required to integrate ICD-11 coding software into their health information management systems (HIMSs) for coding the discharge diagnoses by clinical coders. The ICD-11 coding software used in this study was aligned with the 202105 version of the ICD-11 MMS. The software installation package was distributed to the pilot hospitals for integration into their HIMSs. This integration can be accomplished in 2 ways: either by directly using the provided coding interface or by accessing the application programming interface (API) to develop a custom coding interface tailored to their specific needs. Although a custom coding interface may differ from the provided interface, the principles guiding their coding processes were uniformly instructed as follows: Upon selecting an index term that best matches the clinical diagnostic statement in the ICD-11 coding software, the URI of the ICD-11 Foundation entity that represents the best match term is preserved at the back end. Concurrently, the corresponding ICD-11 MMS stem code is automatically assigned. A comprehensive overview of this stem code is displayed to the coders for verification purposes, including its hierarchy, description, inclusions, exclusions, and coding notes. Where applicable, postcoordination options are presented to facilitate the reporting of more detailed information.

Hospital discharge data, including the ICD-10 and the ICD-11 coded data, generated for 2 consecutive months by participating clinical coders were reported to the National Hospital Quality Monitoring System (HQMS). While the 59 hospitals were tasked with pilot use of the ICD-11 among clinical coders, 14 hospitals were additionally tasked with exploring the use of the ICD-11 among physicians, albeit that is not reported in this article.

A virtual meeting, attended by officials from the National Health Commission and provincial health commissions and focal points from 59 hospitals, launched the program. The meeting disseminated core information about the ICD-11, elucidated its benefits for health data with the goal of reaching a common understanding of the importance of this program, and articulated its tasks and requirements. Subsequently, a virtual technical meeting convened with information technology (IT) personnel from the pilot hospitals and vendors, articulating the requisites for IT infrastructure and providing guidance on integrating the ICD-11 coding software into HIMSs, drawing from relevant experiences of the previous single-center pilot study at PUMCH.

A 2-day online training program on the ICD-11 was conducted on DingTalk (DingTalk Technology, Hangzhou), including 853 participants from the pilot hospitals. The educational materials were developed by referencing the WHO ICD-11 Education Tool and the ICD-11 Reference Guide,^11^^,^^12^ and further refined following the single-center pilot study at PUMCH. The training program covered an overview and basics of the ICD-11, classification hierarchy, coding rules and practical coding scenarios for chapters 1–25, introduction of extension codes, use of the ICD-11 coding software, and a Q&A session. Following the training, 2 exams on the ICD-11 were administered on a web-based platform. Each exam consisted of 13 single-choice questions, 12 true/false questions, and 25 case summaries and diagnostic statements for ICD-11 coding, with each question valued at 2 points for a maximum score of 100 points. Grading was performed instantly post-exam through an integrated algorithm that compared submitted answers with established gold standards. The passing score was determined post-exam based on a review of test performances. Participants who missed or failed the first exam had the chance to take the second exam. A total of 685 participants from 58 hospitals took part in the first exam, and 125 from 38 hospitals participated in the second. Clinical coders from PUMCH acted as trainers in the training program and organized the exams; therefore, they did not participate in the exams. Passing the exam was not a requirement for participating in the 2-month pilot coding, though it was highly recommended.

To provide real-time technical support, collect feedback, and track progress, an online communication mechanism was established using a WeChat (Tencent Technology, Shenzhen) group with all focal points at the pilot hospitals. A separate WeChat group was established among IT experts and personnel from participating hospitals and vendors, to provide technical support for the integration of the ICD-11 coding software.

A series of customized electronic questionnaires were designed using WJX online survey platform (Changsha Ranxing Information Technology, Changsha) and distributed to the focal points, aligning with different stages of the pilot program. The questionnaires are available in a Word format in the Supplementary Material. Feedback on the launch meeting of the pilot program, IT technical meeting, and the training program were gathered with open-ended questions. During the initial phase of the pilot program, questions related to the pilot protocol, integration of the ICD-11 coding software, and coding practices were collected on a weekly basis, and responses to frequently asked questions were provided to the pilot hospitals in the subsequent week. As the majority of the pilot hospitals completed the integration of the ICD-11 coding software, information regarding IT infrastructure, the approach and time for the software integration, technical challenges encountered, and user feedback on the coding software’s functionality and performance were collected.

To ensure the standardized storage and reporting of the ICD-11 coded data by the pilot hospitals, we helped the pilot hospitals verify the test data format post-software integration and provided feedback. A virtual technical meeting was held to provide guidance on encountered issues with the data format, and data samples were provided for reference.

Upon the completion of the requested 2-month coding phase, virtual meetings were conducted with the pilot hospitals. Each hospital presented their work during these meetings and submitted a detailed narrative summary report. As required, the presentation and narrative summary report covered the following areas: accomplishments during the pilot phase; experiences and difficulties encountered; feedback on the overall pilot program; and specific considerations for the ICD-11 implementation, including key factors, critical issues needing resolution, and challenges faced. Key considerations for the ICD-11 implementation were extracted from the detailed narrative reports and categorized using thematic qualitative analysis. An overview of pilot work at each hospital was also provided in a spreadsheet, detailing the number of clinical coders involved, those who passed the ICD-11 exam, and the number of discharges and diagnoses coded with the ICD-11.

### Calculation of the accuracy and intercoder reliability of ICD-11 coding of exam cases

Coding accuracy and intercoder reliability were computed based on the coding results of the 25 case summaries and diagnostic statements provided in the first online exam mentioned above. The analyses were performed to evaluate the effectiveness of the brief training program and inform future improvement, assess the baseline coding competencies of clinical coders before engaging in the pilot coding, and identify the coding variance across the pilot hospitals. The case summaries and diagnostic statements for the ICD-11 coding in the exam were selected by the trainers from a database of coding exercises. Participants were required to assign an ICD-11 MMS code or code cluster that best describes each case summary or diagnostic statement using an ICD-11 coding software embedded in the exam platform. Gold standards for the ICD-11 coding were determined through consensus by 2 clinical coders from PUMCH, with any discrepancies resolved by a third colleague. Some cases allowed for alternative codes (eg, when the code sequence in one cluster does not matter). However, specific rules, such as placing the harm or injury codes before the external cause codes, were maintained. The case summaries and diagnostic statements and the gold standards for the ICD-11 coding are available in the Supplementary Material.

Of the 685 participants from 58 pilot hospitals, 15 were excluded because of missing data in the coding for all 25 cases. To ensure the independence of the coding results of participants within each hospital, one participant was randomly selected from each hospital to generate a subsample for analysis. Consequently, coding results of 58 participants, each representing a different pilot hospital, were used to calculate the coding accuracy and intercoder reliability.

Coding accuracy was calculated in 2 ways: considering the entire coding field (including postcoordination) and considering only one stem code. An entire coding field holds a value of either a single code or a code cluster using postcoordination, as reported by the exam participant for a case. In cases where the gold standard coding requires postcoordination involving multiple stem codes, we exercised careful judgment to determine the appropriate single stem code as being correct (see Supplementary Material). This approach deviates from considering any stem code within the gold standard as correct. For instance, in the coding of “Hemiplegia as a sequela of old cerebral hemorrhage,” the gold standard is denoted as MB53.Z/8B25.1. In our focused analysis, “MB53.Z” alone is identified as the correct stem code. This decision aligns with the ICD-11 guidelines in morbidity coding and the instructions in the training program, which prioritize coding the specific manifestation of the sequela first.^12^

Krippendorff’s alpha was employed to evaluate the intercoder reliability in our study. This measure was calculated considering the entire coding field (including postcoordination). To provide a comprehensive analysis, the Krippendorff’s alpha reliability values were reported in 2 distinct ways: one considering the sequence of the codes, and the other without consideration of the sequence.

Subgroup analyses were performed by categorizing the 25 case summaries and diagnostic statements into 2 groups according to the gold standards of the ICD-11 coding: 7 cases without postcoordination need and 18 cases requiring postcoordination. Coding accuracy and Krippendorff’s alpha were reported for each subgroup.

Statistical analysis was conducted using SPSS version 26.0 (IBM, New York). Krippendorff’s alpha reliability values were computed using the KALPHA macro for SPSS developed by Hayes and Krippendorff, which can be used regardless of the number of coders and levels of measurement and allows for missing data.^13^^,^^14^ Krippendorff’s alpha values range from 0 to 1, with 1 indicating perfect reliability and 0 indicating the absence of reliability. The 95% CI of Krippendorff’s alpha was estimated using the bootstrapping method, with 10 000 bootstrap samples.

### Ethical approval

This study was approved by the Institutional Review Board of PUMCH at the Chinese Academy of Medical Sciences.

## Results

### National pilot study

Of all 59 pilot hospitals, 56 successfully implemented the ICD-11 in morbidity coding. Among the 3 remaining hospitals, 2 integrated the ICD-11 coding software into their systems without the ability to implement the ICD-11 coding in practical scenarios due to insufficient manpower, and 1 dropped out of the program because of difficulties in the transition between an old and a new hospital information system. The profile of the pilot hospitals is presented in Table 1. The HIMSs of the pilot hospitals encompassed both web applications and Windows applications, employing 8 distinct programming languages.

**Table 1.** Hospitals participating in the national pilot program of the ICD-11 morbidity coding.

Hospital type	Recruited hospitals	Included hospitals
	(*N* = 59)	(*N* = 56)
General hospital	34	32
Specialty hospital		
Neoplastic diseases	8	7
Children	6	6
Women and children	4	4
Communicable diseases	2	2
Eye diseases	2	2
Mental health	1	1
Brain diseases	1	1
Cardiovascular diseases	1	1

Based on the feedback from the focal points at pilot hospitals that implemented the ICD-11 morbidity coding, the average time to integrate the ICD-11 coding software was 12.4 days (range: 2-35 days). A total of 53 pilot hospitals integrated the provided coding interface into their HIMSs, with an average integration time of 12.2 days. Four pilot hospitals with the same HIMS vendor used a custom coding interface with provided API, with the integration time ranging from 5 to 30 days. PUMCH was excluded from the calculation because its integration was accomplished in the previous single-center pilot program.

Among the 853 individuals enrolled in the training program, 715 attempted at least one of the exams. For the first and second exams, the passing scores were set at 84 and 80 points, respectively. Specifically, 586 (85.5%) out of 685 participants passed the first exam, and 105 (84.0%) out of 125 participants passed the second. As reported by the focal points at the pilot hospitals, a total of 563 clinical coders engaged in the ICD-11 coding during the pilot program, of which 544 (96.3%) passed the exam. Upon the completion of the pilot program, 3 723 959 diagnoses for 873 425 patients were coded using the ICD-11 MMS, and the coded data were reported to the national HQMS in a standardized format.

The key considerations for the ICD-11 implementation in morbidity coding were extracted from the summary reports submitted by the pilot hospitals and categorized into the following 6 domains (Table 2): ICD-11 content, tools, training, clinical documentation, downstream data utilization, and process and mechanism.

**Table 2.** Key considerations for the ICD-11 implementation.

Category	Key considerations
ICD-11 content	Enrich the ICD-11 Foundation with diagnostic terms and concepts from the ICD-10 Chinese Modification as appropriate
	Increase the number of preconfigured postcoordination options (manually any combinations are possible)
	Define mandatory postcoordination based on country needs
Tools	Increase further the ICD-11 coding efficiency and accuracy (language dependent)
	Create a quality verification tool for the ICD-11 coded data
Training	Establish a systemic training and certification system for trainers and coders
	Develop detailed education materials and coding guidance
	Identify and meet education needs based on an audit of coding data from the pilot program
	Improve data user communication and training to raise awareness of the ICD-11 benefits
	Develop systemic training programs for specialty physicians, eg, in mental health facilities
Clinical documentation	Conduct clinical documentation improvement projects to meet the requirements of details in the ICD-11
	Build the interaction between structured Electronic Medical Records and the ICD-11 or leverage artificial intelligence to facilitate documenting information that should be included when coding
Downstream data utilization	Provide technical guidance for data retrieval and visualization of the ICD-11 coded data
	Enable data utilization for statistics, health care quality surveillance, case mix, etc.
Process and mechanism	Coordinate with various stakeholders to draw a clear roadmap for national implementation
	Determine the duration of the transition phase and prepare resources accordingly
	Conduct pilot studies in resource-limited facilities
	Establish a platform for preliminary assessment of proposals in Chinese submitted by users within the country before they can be translated and submitted to the proposal platform of WHO.

### Coding accuracy and intercoder reliability

The coding accuracy and intercoder reliability for the 25 coding summaries and diagnostic statements and those for the 2 subgroups of cases are presented in Table 3.

**Table 3.** Coding accuracy and intercoder reliability based on the coding results of the ICD-11 exam.

Case summaries and diagnostic statements (*N*)	Coding accuracy		Krippendorff’s alpha	
	Considering the entire coding field (%)	Considering one stem code (%)	Considering the code sequence (95% CI)	Not considering the code sequence (95% CI)
All Cases (25)	82.9	92.2	0.792 (0.788-0.796)	0.799 (0.795-0.803)
Cases without postcoordination need (7)	91.4	93.7	0.852 (0.844-0.858)	0.852 (0.845-0.859)
Cases requiring postcoordination (18)	79.1	91.6	0.761 (0.756-0.766)	0.771 (0.766-0.775)

Across all the coding summaries and diagnostic statements, the overall coding accuracy was 82.9% (range: 15.5%-100%) when considering the entire coding field (including postcoordination) and 92.2% (range: 65.5%-100%) when only one stem code was considered. In the subgroup analysis, the coding accuracy for the 7 cases without postcoordination need was 91.4%, while that for the 18 cases requiring postcoordination was 79.1%, when considering the entire coding field (including postcoordination).

The average occurrence of postcoordination errors was 10.5% (range: 0%-72.4%). Postcoordination errors were categorized as missing (66.4%), incorrect (30.9%), and redundant (2.6%) postcoordination values. Improper use of postcoordination values was observed in 14.1% of the coding results for the cases requiring postcoordination. Incorrect coding results of several selected case are presented in Table 4. Mandatory postcoordination of causing condition was missing in 15.5% and 10.3% in the case of “gastritis due to IgA vasculitis” and “toxic megacolon due to ulcerative colitis,” respectively. The required code for causing condition 8A20 “Alzheimer disease” was missing from 31% of the coding results for “mild dementia caused by late-onset Alzheimer’s disease.” Although postcoordination is deemed redundant when the information has been precoordinated, this is an exceptional case because the code 6D80.1 “Dementia due to Alzheimer’s disease with late onset” should never be used in the primary tabulation and is provided for use as a supplementary code as instructed in the coding note. A coding accuracy of 51.7% was observed in the case of “ototoxic hearing loss caused by gentamicin (normal dose) in therapeutic use,” which involves the utilization of the 3-part (ie, harm-cause-mode) model. Among the 47 participants correctly coding the “harm,” 17 (36.2%) participants either failed in providing the postcoordination value or selected the incorrect value for the “cause” or the “mode.”

**Table 4.** Incorrect coding results of selected cases from the ICD-11 exam.

Case summary or diagnostic statement	Gold standard of the ICD-11 coding	Incorrect coding results (*N*)
Gastritis due to IgA vasculitis	DA42.Y/4A44.92	Missing postcoordination (9) –DA42.Y Incorrect postcoordination (1) –DA42.Y/ME24.Y Incorrect destination stem code (2) –DA42.Z –DA42.Z/4A44.92 Null (1)
Toxic megacolon due to ulcerative colitis	DB32.20/DD71.Z	Missing postcoordination (6) –DB32.20 Incorrect postcoordination (2) –DB32.20/DD71 –DB32.20/DD71.Y
Mild dementia caused by late onset Alzheimer disease	8A20/6D80.1&XS5W, or 6D80.1&XS5W/8A20	Missing postcoordination (19) –6D80.1&XS5W –6D80.1/8A20 Incorrect destination stem code (2) –6D80.Z –6D80.Z&XS5W/8A20 Null (1)
Ototoxic hearing loss caused by gentamicin (normal dose) in therapeutic use (Instruction: please use the 3-part model to indicate harm, cause, and mode)	AB53/PL00&XM3YS5/PL13.2	Missing postcoordination (9) –AB53/PL00 –AB53/PL00&XM3YS5 –AB53/PL13.2 Incorrect postcoordination (8) –AB53&XK9J/NE60 –AB53/NE60 –AB53/NE60&XM3YS5 –AB53/NE60&XM3YS5/PL00 –AB53/NE60&XM3YS5/PL13.2 –AB53/NE60/PL00&XM3YS5 –AB53/PL00&XM3YS5/NE60 Incorrect destination stem code (5) –AB51.1 –PK80.AZ/AB53 –PL00&XM3YS5 –PL00&XM3YS5/AB53 –PL00/AB5Z Null (6)

Overall, Krippendorff’s alpha was 0.792 (95% CI, 0.788-0.796) when the code sequence was considered and 0.799 (95% CI, 0.795-0.803) when the code sequence was not considered. The Krippendorff’s alpha for the subgroup of 18 cases requiring postcoordination reached 0.761 (0.756-0.766) and 0.771 (0.766-0.775) with and without consideration of the code sequence, respectively.

## Discussion

This study reported a large-scale pilot program of the ICD-11 morbidity coding at a national level. The ICD-11 has been successfully implemented in 56 tertiary hospitals in China. Following the pilot program, every pilot region has the required personnel and knowledge to bolster its capacity to promote the implementation of the ICD-11 morbidity coding. Our study detailed the strategies and methodologies used to conduct the program and summarized key considerations for implementing the ICD-11 in morbidity coding from the perspectives of participating hospitals. We expect that other countries can benefit from the results of this study when formulating their own implementation or transition plans.

The integration of the ICD-11 coding software into individual HIMSs is a key premise of success in pilot programs. Integration times varied significantly among the pilot hospitals, ranging from 2 to 35 days. This variation can be attributed to several factors, including the level of expertise and familiarity with the ICD-11 among IT staff, the technical infrastructure, the degree of software compatibility and complexity, and specific customization requirements. It was observed that even among hospitals using the same HIMS vendor, the integration times varied considerably. This suggests that a vendor’s experience in integrating the ICD-11 coding software in one hospital may possibly expedite the integration process in other hospitals. Understanding the IT infrastructure characteristics of all pilot hospitals is necessary to evaluate the applicability of the ICD-11 coding software, and these evaluations should be conducted at the beginning of a pilot program. Since the ICD-11 is entirely digital and the coding frame is different from that of the ICD-10, it is necessary to explain the structure of codes, share a reference format for data storage, and provide a help file to the IT personnel of pilot hospitals and vendors. Communications with IT staff are needed to understand the difficulties and challenges of technical implementation processes. Frequently asked questions and solutions were incorporated in the help file for software integration after the pilot study. It was observed that misinterpretations were frequently generated from the JSON string of the coded data by the IT staff, especially when nested postcoordination was involved. It is helpful to verify the data format of test cases at individual pilot hospitals before the pilot coding phase begins to ensure that the data are correctly stored and reported. The verification of test cases was performed manually in this study; however, we acknowledge that an automatic tool is needed for data verification by hospitals when rolling out the ICD-11 in the country.

The established communication mechanism with focal points was praised by the pilot hospitals, as it enabled timely and ongoing technical support. The feedback from end users likewise provided valuable information for optimizing the experience of using the coding software, enhancing the ICD-11 content in terms of the classification and coding rules, and identifying the needs for future training and education. According to the feedback from the focal points of the individual hospitals, a few perspectives should be considered regarding the ICD-11 implementation, including the ICD-11 content, tools, training, clinical documentation, downstream use, and process and mechanism.

One aspect concerns the representation of concepts in the ICD-10 Chinese Modifications with the ICD-11. The ICD-11 is a regularly updated classification and terminology system, which evolves along with the development of medical science and terminology. It benefits from the input of global users, provided through the open proposal platform. During and after the pilot study, we submitted proposals to the ICD-11 proposal platform to add needed concepts to the ICD-11 and to reduce the need for manual postcoordination. We have established a national platform to collect proposals in Chinese, the language in which all Chinese clinical coders are proficient. It is designed for preliminary assessment of proposals. Proposals that have merit will be translated into English and submitted to the WHO Proposal Platform for consideration. This mechanism is anticipated to enhance the active involvement of Chinese contributors in the ongoing maintenance of the ICD-11.

Postcoordination extensively increases the capacity of the ICD-11 to represent clinical information^15–17^; however, the potential decrease in productivity due to manual postcoordination was also considered. All the concepts in the ICD-10 national modification are in the precoordinated manner, many of which are represented via postcoordination in ICD-11. Studies have reported positive outcomes in representing ICD-10-CM (US modification) concepts with the ICD-11 postcoordination.^9^ The ICD-11 Coding Tool developed by the WHO has the capability to return results from code clusters, even when these clusters are not individual entities in the classification or in the foundation. This is sometimes referred as “virtual index.” For example, the Coding Tool provides “2C6Z&XK8G” when searching for “left breast cancer.” This has substantially reduced the effort of selecting postcoordination values. From the perspective of a given country, equivalence bridges between concepts included in the ICD-10 national modifications and the ICD-11 code clusters might serve as a kind of “virtual index.” This means that searching for a specific ICD-10 national modification concept, which requires representation with ICD-11 postcoordination, could result in a given ICD-11 code cluster. It was raised that the requirements for mandatory postcoordination should be determined based on the level of details required in data reporting of individual countries. Although no national modifications have been generated from the common core of the ICD-11, the potential of the ICD-11 to satisfy country needs is ensured by the WHO with maintenance policies and technical mechanisms.^18^ In addition, a few suggestions for improving coding efficiency and accuracy were received from pilot hospitals, including predefining code clusters for frequently used diagnostic terms, constructing specialty-specific diagnostic libraries, improving search functions based on user feedback, further adapting the coding process to the environment of the individual HIMSs, and developing quality verification tools for the ICD-11 coded data.

Systemic training and certification systems for trainers and coders could ensure the presence of a qualified workforce and the recording of reliable data. Countries have developed specific coding instructions to ensure consistency in the ICD-10 coding and data usage, many of which should be reflected in the ICD-11 coding guidelines. The coding instructions will likely be further refined based on the input received from this pilot program and preliminary assessment of the coded data. For example, the requirement for postcoordination of external cause when multiple injuries occur and each injury being coded separately should be clarified, since it required substantial effort from the coders in our pilot study to repeat the external cause code as instructed at the postcoordination area of each injury code.

Another challenge is to identify and fill the gaps between current clinical documentation and the desired documentation for the ICD-11 coding. Feedback from some focal points at the pilot hospitals revealed challenges faced by clinical coders in easily finding the desired details within diagnostic statements or throughout medical records. The ICD-11, with its alignment to current medical terminologies, incorporation of more fine-grained concepts, and postcoordination options for capturing additional details, may necessitate a higher level of detail in clinical documentation than what was previously required. Health care institutions will need to assess and improve their clinical documentation for the ICD-11 coding. The interaction between electronic medical records and the ICD-11 might be explored to facilitate the identification of information that should be coded. It is expected that automated clinical coding using the ICD-11 and the improvement of clinical documentation will benefit from the application of artificial intelligence. The necessity of providing training for mental health professionals to use the ICD-11 Clinical Descriptions and Diagnostic Requirements (CDDR) was highlighted, because the classification of mental, behavioral, or neurodevelopmental disorders underwent substantial revisions from the ICD-10 to the ICD-11; the need for training has been recognized by the mental health society and will be the focus of work in the next phase of piloting implementation in China.^19–21^

The ICD-11 coded data were generated and reported in this pilot program; however, downstream data applications have yet to be established. The added value of the ICD-11 coding to health care is most obvious when these data are used to generate insights and inform decision making. Further communication with and training of data users is necessary to raise awareness and motivate exploring the data application. It is essential to coordinate with various stakeholders to draw a clear roadmap and clarify the duration of the transition phase so that resources can be properly organized and prepared.

The digital ICD-11 system can ensure good coding quality at a lower training cost. The exam results indicated that a 2-day training program sufficed to achieve an accuracy rate of 82.9% when considering the entire coding field (including postcoordination) and 92.2% when only one stem code was considered, which are higher than the ICD-11 accuracy rates reported by Lee (71.6%) and Zarei (74.2%).^22^^,^^23^ Krippendorff’s alpha reliability values of 0.792 overall and 0.761 for the cases requiring postcoordination were achieved in the ICD-11 coding exam following the training program, better than that reported by Eisele (0.672 for 4-character codes and 0.251 for all characters).^24^ The differences in coding accuracy and intercoder reliability between our study and other studies may be explained by the differences in the selected diagnostic statements for assessment, the level of training provided, and the tool environment for coding. Results of the subgroup analyses suggest that the complexity added by postcoordination impacts coding accuracy and intercoder reliability.

Improper use of postcoordination was found in 10.5% of the coding results. This underscores the imperative for additional training on postcoordination, with anticipated improvement of coding accuracy and intercoder reliability following the training. In the case of “gastritis due to IgA vasculitis” and “toxic megacolon due to ulcerative colitis,” where postcoordination for causing condition is mandatory at the stem code, it was observed that over 10% of the exam participants did not provide the required postcoordination value in their coding results. This oversight may have been influenced by the coding software’s search results, which displayed an exact match term. This could have potentially led the participants to mistakenly believe that selecting the exact match term alone was sufficient, without need for further postcoordination to the related stem code. However, if postcoordination is indicated as optional, it relies on the data types that are utilized in data analysis to decide whether postcoordination is required or whether the URI of the best match term is sufficient, which should be clarified in the protocols of pilot studies.

The 3-part model is a new feature of the ICD-11 and provides an innovative method of capturing patient safety events.^8^^,^^25–27^ In one case of the exam, participants were instructed to apply the 3-part model; this resulted in a low accuracy rate of 51.7%. A considerable diversity of coding results was observed in this case among the 58 exam participants. This highlights the future need for reinforced education on the utilization of the 3-part model in the training program, which is essential to ensure precise and comprehensive patient safety surveillance. Further investigation is warranted to assess the accuracy and understand the challenges of applying the 3-part model, using a broader range of case scenarios.

There are limitations and lessons learned from this pilot study. First, the pilot study exclusively involved leading hospitals in each region, with robust IT infrastructure and adequate supporting resources. Future pilot studies should encompass facilities with limited resources, to better understand the feasibility and challenges regarding the nationwide implementation of the ICD-11. Second, due to absence of a feature in the HIMSs of pilot hospitals that allows the selection of a main condition in the ICD-11 coding distinct from the ICD-10 coding, coders were instructed to adhere to the rule set from the ICD-10 in case of any inconsistencies. While this ensured that routine statistics and reimbursement using the ICD-10 coded data were not affected, it may lead researchers to overlook the impact on morbidity during the transition from the ICD-10 to the ICD-11. It is suggested a functionality be designed to allow the independent selection of main conditions using the ICD-11 in future pilot programs. Third, the coding accuracy and reliability reported in this article were derived from an ICD-11 coding exam, rather than from an assessment using the real-world data generated from the pilot program. The exam cases, being somewhat controlled and limited in scope, may not encompass the full spectrum of challenges and variables present in real-world coding scenarios. Consequently, the accuracy and reliability in real-world practice could differ from the figures reported in the exam context. This anticipated discrepancy underscores the necessity of conducting an audit of the real-world data. The results of an audit program of the ICD-11 coded data in PUMCH will be reported in a separate paper. Fourth, the difference in coding productivity between the ICD-10 and the ICD-11 was not compared in this study. A well-designed dual coding study with a controlled sample size could be conducted to evaluate the workforce requirements and training needs of implementing the ICD-11. Fifth, it is imperative to address gaps identified in the mapping or content coverage evaluation between ICD-10 national modifications and the ICD-11 before pilot studies are conducted. This effort would significantly increase the satisfaction of coders who are familiar with the ICD-10 national modifications. Last, it is noteworthy that the ICD-11 coding software utilized in our study did not incorporate the sanctioning rules which are functioning in the WHO ICD-11 Browser and Coding Tool. These rules can improve coding accuracy by preventing incorrect or prohibited postcoordinations. However, we conducted a thorough review of the coding errors made by the 58 coders in the subsample, cross-referencing with the WHO ICD-11 Browser, and revealed that the absence of sanctioning rules did not affect the coding accuracy in the exam. Future research could investigate the impact of sanctioning rules on coding accuracy.

## Conclusions

This nationwide pilot study has enhanced national technical readiness for the ICD-11 implementation in morbidity, elucidating the key factors that should be carefully considered in future endeavors. The good accuracy and intercoder reliability of the ICD-11 coding achieved following a brief training program underscore the potential for the ICD-11 to reduce training costs and provide high-quality health data. Further assessments of the ICD-11 coding quality and efficiency in the real-world practice are warranted to identify the areas for improvement and plan for coding workforce in the transition to the ICD-11. Experiences and lessons learned from this study have contributed to WHO’s work on the ICD-11 and can inform other countries when formulating their transition plan.

## Supplementary Material

ocae031_Supplementary_Data

## Data Availability

The case summaries and diagnostic statements, the gold standards and coding results used for the analysis of the ICD-11 coding accuracy, and intercoder reliability are available in the Supplementary Material.
