# Supplementary material for: Methodologies and key considerations for implementing the International Classification of Diseases-11th revision morbidity coding: insights from a national pilot study in China
Source: J Am Med Inform Assoc. 2024 Mar 1;31(5):1084–92. doi: 10.1093/jamia/ocae031 (PMC11031236; doi:10.1093/jamia/ocae031)
Supplement: ocae031_Supplementary_Data [file ocae031_supplementary_data.zip › ocae031_Supplementary_Data/Questionnaires to collect feedback in the ICD-11 pilot program.docx]

A series of customized electronic questionnaires were designed using WJX online survey platform and distributed to the focal points, aligning with different stages of the pilot program. The questionnaires are originally in Chinese in electronic format. We have translated the questionnaires into English and presented them in Word format.

**Feedback on ICD-11 Pilot Program Series Meetings**

Please fill out your feedback on the launch meeting of the ICD-11 pilot program, the IT technical meeting, and the ICD-11 training program.

**1. Full name of your hospital *(required)**
_________________________________

**2. Please summarize and submit your hospital's feedback (optional)**

**I. What questions or comments do you have about the information (e.g. pilot plan, task, timeline) delivered in the launch meeting of the pilot program?**

(1) ______________________________________________________________
 (2) ______________________________________________________________
 (3) ______________________________________________________________
 (4) ______________________________________________________________
 (5) ______________________________________________________________

Add more content here______________________________________________

**II. What questions or comments do you have about the information (e.g. system integration approach, coding process, data format) communicated in the IT technical meeting?**

(1) ______________________________________________________________
 (2) ______________________________________________________________
 (3) ______________________________________________________________
 (4) ______________________________________________________________
 (5) ______________________________________________________________

Add more content here______________________________________________

**III. What questions or comments do you have about the ICD-11 training program?**

(1) ______________________________________________________________
 (2) ______________________________________________________________
 (3) ______________________________________________________________
 (4) ______________________________________________________________
 (5) ______________________________________________________________

Add more content here______________________________________________

**Feedback on ICD-11 Pilot Work Progress**

Please provide the progress and issues of the ICD-11 pilot work in your hospital this week. (*: Indicates the field is required to be filled in)

**1. Full name of your hospital ***
_________________________________

**2. What is the progress of the ICD-11 pilot work in your hospital? [Multiple choice] ***
 □ Initiated, preparing software and hardware and other basic conditions
 □ Completed preparation of software and hardware and other basic conditions
 □ Completed integration of ICD-11 with hospital information management systems
 □ Started ICD-11 coding work by clinical coders
 □ Other conditions to be explained _________________*

**3. What questions or suggestions do you have about the ICD-11 pilot protocol? [Multiple choice] ***
 *Check the box before the number, then fill in the blank with specific questions or suggestions. Please at least check one.*
 □ None
 □ 1. _________________*
 □ 2. _________________*
 □ 3. _________________*
 □ 4. Other _________________*

**4. What questions or suggestions do you have about the ICD-11 software integration? [Multiple choice] ***
 Check the box before the number, then fill in the blank *with specific questions or suggestions*. Please at least check one.
 □ None
 □ 1. _________________*
 □ 2. _________________*
 □ 3. _________________*
 □ 4. Other _________________*

**5. What questions do you have about ICD-11 coding? [Multiple choice] ***
 Check the box before the number, then fill in the blank *with specific questions or suggestions*. Please at least check one.
 □ None
 □ 1. _________________*
 □ 2. _________________*
 □ 3. _________________*
 □ 4. Other _________________*

**ICD-11 Pilot Hospital Software Integration Survey**

Please have the focal point at the pilot hospital, together with the person in charge of the hospital's informatics department, fill in this survey. (*: Indicates the field is required to be filled in)

**1. Full name of your hospital ***
_________________________________

**2. Main types and proportions of client operating systems in your hospital [Multiple Choice]***
 □ Windows XP; Proportion: ____%
 □ Windows 7; Proportion: ____%
 □ Windows 10; Proportion: ____%
 □ Other ___________________; Proportion: ____%

**3. Type of the Hospital Information Management System (HIMS) in your hospital [Single Choice] ***

○ Windows application

○ Web application, please specify required browser (including the version):________________

**4. Programming Language (including the version) of HIMS in your hospital***

_________________________________

**5. Approach of integration of the ICD-11 coding software in the HIMS [Single choice] ***

○ Use the provided coding interface

○ Develop custom coding interface with provided API

**6. How is the progress of the ICD-11 pilot work? [Single Choice] ***

○ Not yet completed the integration of ICD-11 coding software in the: ___________________*

*Please fill in the blank the reason for the incomplete integration and the expected date of completion*

○ ICD-11 coding software integration has completed, while formal coding not started: ___________________*

*Please fill in the blank the time taken by the system vendor to complete the integration (in working days)*

○ ICD-11 coding work has started:

___________________*

*Please fill in the blank the time taken by the system vendor to completion of the integration (in working days)*

**7. Are there any technical challenges during the integration process with the vendor? Please specify. [Multiple Choice] ***

*Check the box before the number, then fill in the blank to specify the issues. Please at least check one.*
 □ None
 □ 1. _________________*
 □ 2. _________________*
 □ 3. _________________*
 □ 4. _________________*

**8. What are your comments or suggestions on the current coding software? [Multiple Choice] ***

*Check the box before the number, then fill in the blank to specify your comments or suggestions. Please at least check one.*
 □ None
 □ 1. _________________*
 □ 2. _________________*
 □ 3. _________________*
 □ 4. _________________*

**Feedback on ICD-11 Coding Issues**

Please fill in the coding-related issues and suggestions you encountered in this week's pilot work. (*: Indicates that the field is required to be filled in)

**1. Feedback 1**

_________________________________

**2. Feedback 2**

_________________________________

**3. Feedback 3**

_________________________________

**4. Feedback 4**

_________________________________

**5. Feedback 5**

_________________________________

**6. Other feedback**

_________________________________

**7. Full name of your hospital ***

_________________________________
